# Supplementary material for: Realizing self-sustained biomass gasification in a lab-scale downdraft reactor for compact CHP applications
Source: PLoS One. 2026 Feb 27;21(2):e0343490. doi: 10.1371/journal.pone.0343490 (PMC12948118; doi:10.1371/journal.pone.0343490)
Supplement: S1 Table — (DOCX) [file pone.0343490.s001.docx]

**S1 Table.** Molar fraction and molar heat capacities of the syngas components at ER = 0.60, used for calculating the mixture heat capacity.

| Component | Molar fraction | Heat capacity (J mol^−1^ K^−1^) |
| --- | --- | --- |
| CO | 0.152 | 29.8 |
| CO_2_ | 0.065 | 54.3 |
| H_2_ | 0.05 | 30.2 |
| CH_4_ | 0.002 | 54.0 |
| O_2_ | 0.059 | 34.9 |
| N_2_ | 0.672 | 32.7 |
